# ARHGEF5 binds Drebrin and affects α-tubulin acetylation to direct neuronal morphogenesis and migration during mouse brain development

**DOI:** 10.3389/fnmol.2024.1421932

**Published:** 2024-06-12

**Authors:** Ji-ye Kim, Hee-Gon Hwang, Hye-Jin Jeon, Seung Il Kim, Min-kyu Kim, Jeong-Yoon Kim

**Affiliations:** ^1^Department of Microbiology and Molecular Biology, Chungnam National University, Daejeon, Republic of Korea; ^2^Digital Omics Research Center, Korea Basic Science Institute, Cheongju, Republic of Korea; ^3^Divison of Animal and Dairy Science, Chungnam National University, Daejeon, Republic of Korea

**Keywords:** ARHGEF5, Drebrin, microtubule, neuronal morphogenesis, neuronal migration

## Abstract

Rho guanine nucleotide exchange factors (Rho GEFs) activate Rho GTPases, which act as molecular switches regulating various essential cellular functions. This study investigated the role of ARHGEF5, a Rho GEF known for its involvement in cell migration and invasion processes, in the context of brain development. We found that ARHGEF5 is essential for dendrite development during the early stages of neuronal growth. We also discovered that ARHGEF5 binds to Drebrin E, which is vital for coordinating actin and microtubule dynamics, and facilitates the interaction between Drebrin E and Cyclin-dependent kinase 5, which phosphorylates Drebrin E. Notably, ARHGEF5 deficiency resulted in a decrease in acetylated α-tubulin levels, and the expression of an α-tubulin acetylation mimetic mutant (K40Q) rescued the defects in dendrite development and neuronal migration, suggesting ARHGEF5’s role in modulating microtubule stability. Additionally, ARHGEF5 was shown to influence Golgi positioning in the leading processes of migrating cortical neurons during brain development. Our study suggests that ARHGEF5 plays a crucial role in integrating cytoskeletal dynamics with neuronal morphogenesis and migration processes during brain development.

## 1 Introduction

Rho guanine nucleotide exchange factors (Rho GEFs) stimulate the exchange of GDP for GTP to generate the active form of Rho GTPases, which act as molecular switches to regulate actin organization, cell motility, polarity, growth, survival, and gene expression (Bos et al., 2007; Hodge and Ridley, 2016). The human genome encodes over 70 Rho GEFs, each displaying specificity towards distinct members of the Rho GTPase family such as RhoA, Rac1, and Cdc42 (Rossman et al., 2005). This specificity is crucial for the precise spatial and temporal regulation of signaling pathways to ensure accurate cellular responses to physiological and environmental changes. The central role of Rho GEFs in managing key cellular functions through Rho GTPases means that their dysregulation is associated with various pathologies, including cancer, neurological disorders, and cardiovascular diseases, emphasizing the importance of their regulation for cellular homeostasis and health (Cook et al., 2014).

Among the Rho GEF proteins, ARHGEF5 is recognized for its role in dendritic cell migration and Src-induced podosome formation, highlighting its significant impact on actin cytoskeleton reorganization (Wang et al., 2009; Kuroiwa et al., 2011). Furthermore, ARHGEF5’s critical involvement in the metastatic behavior of human colorectal cancer cells (HCT116) underscores its function in cancer progression (Mihira et al., 2012; Komiya et al., 2016). These observations emphasize ARHGEF5’s importance in cell migration and invasion processes. Additionally, the loss of Arhgef5 in mouse skeletal muscles leads to defects in the neuromuscular junction integrity, characterized by increased fragmentation of the postsynaptic apparatus, possibly due to the abnormal function of the GTPases RhoA and Cdc42 (Bernadzki et al., 2020). Despite its established roles in skeletal muscle, immune, and cancer cell contexts, ARHGEF5’s function during brain development remains largely unexplored. Given the integral role of Rho GEFs in modulating cytoskeletal dynamics, which is essential for neuronal migration and morphogenesis (Dent and Kalil, 2001; Schaar and Mcconnell, 2005), ARHGEF5’s potential contribution to brain development warrants thorough investigation.

In this study, we demonstrate that ARHGEF5 deficiency caused impairment in dendrite morphogenesis and neuronal migration during cortex development, indicative of the indispensable role of ARHGEF5 during brain development. We identified Drebrin, which connects F-actin with microtubules, as a novel interactor of ARHGEF5. Furthermore, we provide evidence that ARHGEF5 is involved in α-tubulin acetylation, suggesting a novel mechanism through which ARHGEF5 coordinates cytoskeletal dynamics.

## 2 Materials and methods

### 2.1 Animals

All animal studies were approved by the Institutional Animal Care and Use Committee of Chungnam National University. For culturing primary neurons, pregnant SD rats were purchased from DBL (Eumseong, South Korea). For *in utero* electroporation, pregnant ICR mice were used. Mice were housed in groups (4–5 mice per group) under a 12 h light–dark cycle with *ad libitum* access to food and water. For timed mating, the day when vaginal plug appeared was designated as embryonic day 0.5 (E 0.5).

### 2.2 Plasmids

For expression of ARHGEF5, Drebrin E, and CDK5, their coding regions were amplified by PCR, and subcloned into pRK5-HA and pEBG-GST vectors. Tublin K40Q expression vector was a gift from Kenneth Yamada (Addgene plasmid #105302). ARHGEF5 shRNA expression vector was constructed using pSUPER vector system (Oligoengine) according to the manufacturer’s recommendations. The sequences of targets were as follows:

Human ARHGEF5 shRNA 5’-GCAACATGACAAACTTCCTAT-3’.
Mouse, rat ARHGEF5 shRNA 5’-GGAAGAATGGCTAGACATA-3’.

For visualization of transfected neurons, shRNA-expressing cassettes were subcloned into pCAGGS-tdTomato. For visualization of the Golgi apparatus, Venus-tagged GalT2 was subcloned into pCAGGS vector.

### 2.3 *In utero* electroporation

Timed pregnant ICR mice were used for *in utero* electroporation as previously described (Kim et al., 2020). Briefly, at embryonic day 14.5 (E 14.5), dams were anesthetized by an intramuscular injection of zoletil/xylazine mixture. The embryos were intracerebrally injected with 2 μg of plasmid DNA (rodent ARHGEF5 shRNA for the knockdown group and the pSUPER vector for the control group) mixed with 0.1% fast green dye (Carl Roth) using a pulled glass microcapillary (World Precision Instruments). After injection, electroporation was conducted with a forceps-type electrode targeting the cerebral cortex. Four 33-V pulses with a duration of 50 ms were applied at 950-ms intervals with an electroporator (NEPA 21). After the electroporation, the uterus was put back into the abdominal cavity, and the abdominal wall and skin were sutured to allow the embryos to develop until embryonic day 18.5 (E 18.5). At E 18.5, brains were fixed in 4% paraformaldehyde with 0.12 M sucrose in PBS. Coronal brain sections were obtained using Cryostat (Leica).

### 2.4 Cell culture and transfection

#### 2.4.1 HEK 293 T cells

HEK 293 T cells (human embryonic kidney cell line) were cultured in Dulbecco’s medified Eagle’s Medium (DMEM) (Gibco) with 10% fetal bovine serum (Gibco), penicillin–streptomycin (Gibco) at 37°C in humidified atmosphere with 5% CO_2_. Cells were transfected by using PEI reagent (Sigma) according to the manufacturer’s instructions.

#### 2.4.2 Hippocampal neurons

Primary hippocampal neurons were prepared from rat embryos (E 18.5) as previously described (Kaech and Banker, 2006). Briefly, the hippocampus was dissected from rat embryos, trypsinized, and triturated through a glass Pasteur pipette. Dissociated neurons were plated at a density of 250,000 neurons per 4 pieces of glass coverslip previously coated with 1 mg/mL poly-L-lysine (Sigma) and cultured in glial cell co-culture system. The cells were maintained in Neurobasal medium (Gibco) supplemented with B27 (Gibco), 1 × GlutaMAX (Gibco). Neurons were transfected using electroporator (NEPA21) according to the manufacture’s recommendation. Neurons were fixed after 6 days *in vitro* (DIV) in 4% paraformaldehyde with 0.12 M sucrose in PBS for 15 min at room temperature.

#### 2.4.3 Cortical neurons

Cerebral cortical regions were isolated from rat embryos, and dissociated with 0.25% trypsin digestion. Cortical neurons were plated in the 100 mm dishes coated with 0.1 mg/mL poly-L-lysine (Sigma). The cells were maintained in Neurobasal medium (Gibco) supplemented with B27 (Gibco), 1 × GlutaMAX (Gibco). Cortical neurons were transfected before plating by electroporator (NEPA21).

### 2.5 ARHGEF5 interacting protein pull-down assay

*E.coli* cells carrying a plasmid expressing GST-fused ARHGEF5 protein were cultured to a density of OD_600_ = 2.0. Cells were cultured at 20°C overnight by adding 0.1 mM isopropyl-β-D-thiogalactopyranoside to culture medium for induction of fusion proteins. *E.coli* cells were harvested by centrifugation at 3,000 rpm., 4°C for 10 min, and the pellet resuspended in lysis buffer (20 mM HEPES (pH 7.5), 150 mM NaCl, 5 mM MgCl_2_, 1% Triton X-100, 1 mM DTT, 10 mM NaF, 1 mM PMSF) was sonicated on ice. After sonication, the sample was centrifugated at 13,000 rpm., 4°C for 10 min. The supernatant was incubated with Glutathione sepharose bead (GE Healthcare) at 4°C for 1 h with rotation. The beads were washed three times with lysis buffer. And rat embryo brains (embryonic day 19) were lysed with lysis buffer. The lysates were cleared by centrifugation at 13,000 rpm., at 4°C for 25 min. GST-ARHGEF5 protein-bound beads were incubated with rat embryo brain lysates at 1 ug beads per 1 mg lysates and rotated for 45 min, at 4°C. Beads were washed three times with lysis buffer and resuspended in 5 × Laemmli sample buffer. After SDS-PAGE running, separated proteins were stained using silver staining kit (ELPIS biotech) according to the manufacturer’s recommendation. Bands were excised and digested with trypsin. Peptides were analyzed by LC–MS and Mass Spectrometer. Resultant MS–MS spectra were searched using the MASCOT program against the NCBI Data Base.

### 2.6 GST pull-down assay

HEK 293 T cells were co-transfected with expression vectors. Cells were lysed in ice-cold lysis buffer (20 mM Tris (pH 7.4), 1 mM EDTA, 150 mM NaCl, 10% Glycerol, 1% NP-40, 10 mM NaF, 1 mM Na_3_VO_4_, 1 mM PMSF). Total cell lysates centrifugated at 13,000 r.p.m., for 30 min at 4°C. The whole cell lysates were incubated at 4°C with glutathione sepharose bead (GE Healthcare) for 6 h ~ overnight. Beads were washed three times with 1 × PBS and resuspended in 2 × Laemmli sample buffer. Proteins were separated by SDS-PAGE and detected by immunoblots with antibodies.

### 2.7 RhoA activity assay

RhoA activity assay was conducted as previously described (Guilluy et al., 2011). HEK 293 T cells or cortical neurons were co-transfected with expression vectors. Cells were lysed in ice-cold RBD buffer (50 mM Tris (pH 7.5), 500 mM NaCl, 2% NP-40, 10 mM MgCl_2_, 1 mM DTT, 10 mM NaF, 1 mM PMSF). Total cell lysates were cleared by centrifugation at 13,000 rpm, 4°C for 30 min. The whole cell lysates were incubated at 4°C for 1 h with GST-RBD (Rho-binding domain from Rhotekin) made by *E.coli* lysates. Beads were washed once with wash buffer (25 mM Tris (pH 7.5), 50 mM NaCl, 30 mM MgCl_2_, 1 mM DTT, 10 mM NaF, 1 mM PMSF) and resuspended in 2 × Laemmli sample buffer. Bound RhoA was separated by 12% SDS-PAGE and detected by immunoblot with antibody against RhoA.

### 2.8 Immunoblot

Prepared samples were loaded onto 8–12% SDS-PAGE gels, run at 65-95 V, and then transferred onto polyvinylidene fluoride (PVDF) membranes (Merck) at 50–60 V for 2 h. The membranes were blocked with 5% skim milk in Tris-buffered saline (TBS) containing 0.05% Tween 20 and probed with diluted primary antibodies overnight at 4°C. The following antibodies were used: anti-ARHGEF5 (1:1000, Proteintech), anti-Drebrin (1:1000, Abcam), anti-HA (1:5000, Santacruz Biotechnology), anti-GST (1:5000, Sigma), anti-β-actin (1:5000, Sigma), anti-RhoA (1:3000, Cell Signaling Technology), anti-GAPDH (1:3000, Invitrogen), anti-α-tubulin (1:5000, Santacruz Biotechnology), and anti-acetyl-α-tubulin Lys40 (1:5000, Cell Signaling Technology). Membranes were washed five times with 1 × TBST and incubated at room temperature for 2 h with appropriate secondary antibodies (1,10,000, Thermo). The membranes were washed five times with 1 × TBST and incubated in ECL solution (Thermo). Blots were detected using Chemidoc (Biorad).

### 2.9 RNA isolation, cDNA synthesis, real-time PCR

Total RNA was purified with the NucleoSpin RNA Kit (MN) according to the manufacturer’s recommendations. cDNA was synthesized using ReverTra Ace qPCR RT kit (Toyobo) and analyzed by quantitative RT-PCR. The real time PCR was performed with RealHelix qPCR Kit (NanoHelix) and CFX connect system (Biorad). Relative expression levels (normalized to GAPDH) were determined using the comparative C_T_ method (2^-ΔΔCt^).

### 2.10 Image acquisition

Images were obtained using an Olympus BX51 microscope (DAPI excitation and emission, 330–385 nm and 420 nm; GFP excitation and emission, 460–490 nm and 520 nm; RFP excitation and emission 530–550 nm and 575 nm) with 40x objective (4,080 × 3,072 pixels), and a Carl Zeiss LSM 880 with an Airyscan confocal microscope (DAPI excitation and emission, 405 nm and 444 nm; GFP excitation and emission, 488 nm and 522 nm; RFP excitation and emission, 561 nm, 604 nm) with 20x objective and 40x objective (2,048 × 2,048 pixels, 1 μm z-stack interval). Images were modified using Image J.

### 2.11 Statistics

All data are presented as mean ± standard error of the mean (S.E.M.). Unpaired, two-tailed Student’s t-test was used to compare two groups, and one-way ANOVA with Tukey’s post-hoc test was used for comparison between multiple groups. *p* values ≤0.05 were considered statistically significant and marked as “*.” p values ≤0.01 were marked as “**” and p values ≤0.001 were marked as “***.”

## 3 Results

### 3.1 ARHGEF5 is expressed during the early brain development stage

**Figure 1:**
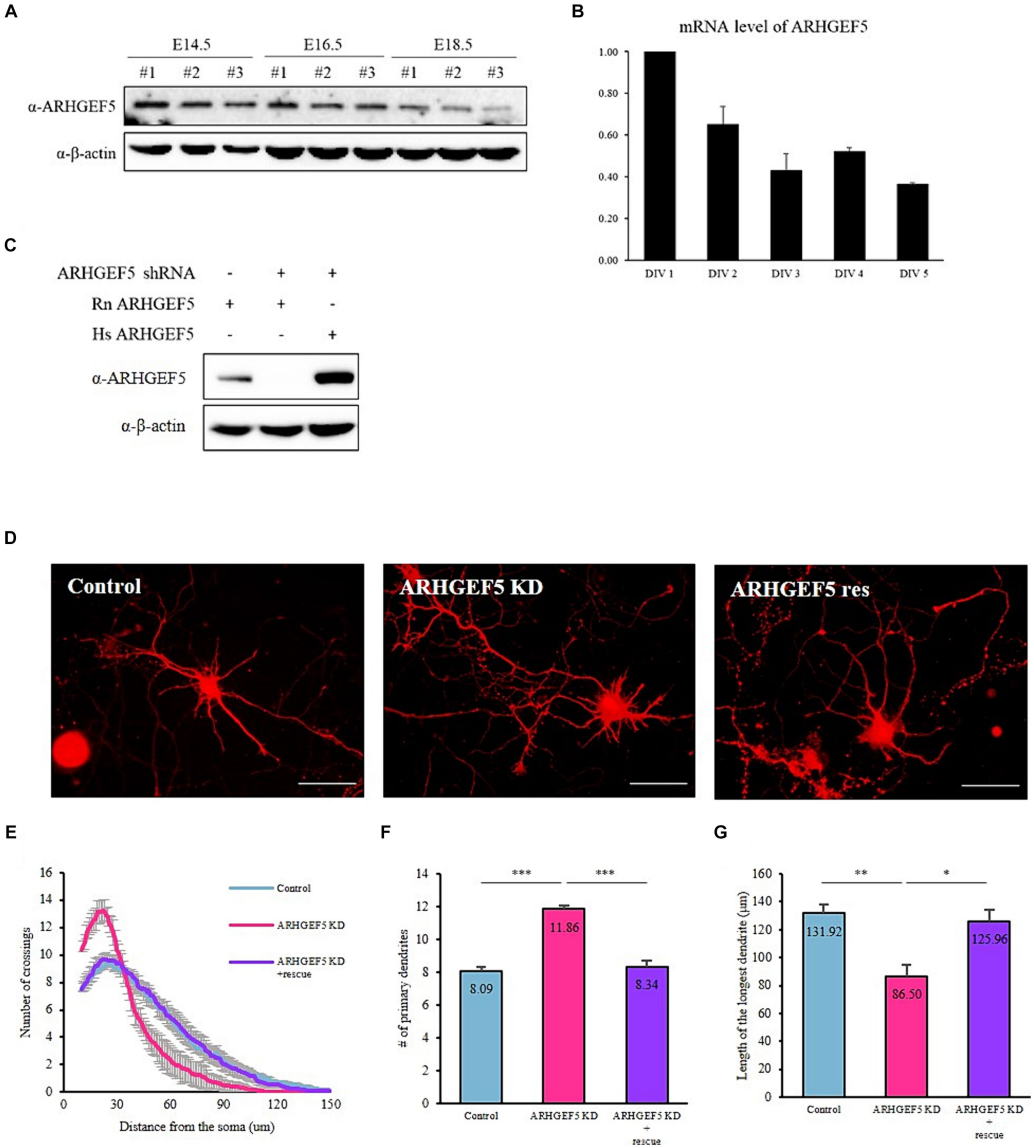
ARHGEF5 is expressed during the early stages of brain development **(A)** Immunoblot shows the expression levels of ARHGEF5 in embryonic mouse brain lysates. β-Actin was used as the loading control. **(B)** A bar graph showing the relative mRNA expression levels of ARHGEF5 in cultured rat cortical neurons. **(C)** Immunoblot shows the shRNA knockdown efficiency in HEK293T cells transfected with rodent ARHGEF5-targeting shRNA, rodent ARHGEF5 construct, and human ARHGEF5 construct. β-Actin was used as the loading control. **(D)** Representative images of rat hippocampal neurons transfected with ARHGEF5 shRNA or ARHGEF5 shRNA plus shRNA-resistant ARHGEF5 construct. **(E)** Quantification of dendritic complexity using Sholl analysis. **(F)** Number of primary dendrites. **(G)** Length of the longest dendrite. *n* > 40 from four independent experiments for each group. All data are presented as mean ± SEM. ^*^p < 0.05, ^**^p < 0.01, ^***^p < 0.001 determined by one-way ANOVA with Tukey’s *post hoc* test.

To investigate the potential role of ARHGEF5 in the central nervous system, we looked for the expression pattern of ARHGEF5 in an atlas^1^ and found that ARHGEF5 is expressed at relatively low levels and exhibits low regional specificity in both the human and mouse brains. Then, we analyzed the expression pattern of ARHGEF5 during brain development. In the mouse cortex, ARHGEF5 protein levels gradually decreased during the embryonic stage (Figure 1A). Moreover, in cultured cortical neurons, ARHGEF5 mRNA expression levels also declined progressively from DIV 1 to DIV 5 (Figure 1B). These data suggest that ARHGEF5 is likely to function in the early stage of cortical neuron development.

Subsequently, we examined the impact of ARHGEF5 deficiency on the morphological development of cultured neurons. The efficiency of shRNA-mediated knockdown and the expression of shRNA-resistant ARHGEF5 were verified in HEK293T cells transfected with rodent ARHGEF5-targeting shRNA, rodent ARHGEF5 construct, and human ARHGEF5 construct (shRNA-resistant ARHGEF5) (Figure 1C). Knockdown of ARHGEF5 led to the emergence of neurons characterized by shorter, more numerous primary dendrites than control neurons. This morphological defect was rescued by co-transfection with shRNA-resistant ARHGEF5 (Figures 1D–G). This finding implies a potential role for ARHGEF5 in the regulation of cytoskeletal elements critical for the proper development of dendrites.

### 3.2 ARHGEF5 interacts with Drebrin in the embryonic brain

We sought to identify ARHGEF5 interactors in the embryonic brain to investigate the molecular mechanism underlying ARHGEF5’s function during neuron development. We expressed GST-tagged ARHGEF5 in *E. coli* and used it for protein–protein interaction assays with proteins extracted from rat embryonic brain tissues. This strategy enabled us to identify Drebrin E as a novel ARHGEF5 interactor (Supplementary Figure S1). Drebrin organizes F-actin bundles and facilitates interactions between F-actin and microtubules, thereby playing a critical role in neuronal morphogenesis and migration through its modulation of cytoskeletal dynamics (Geraldo et al., 2008; Dun et al., 2012).

**Figure 2:**
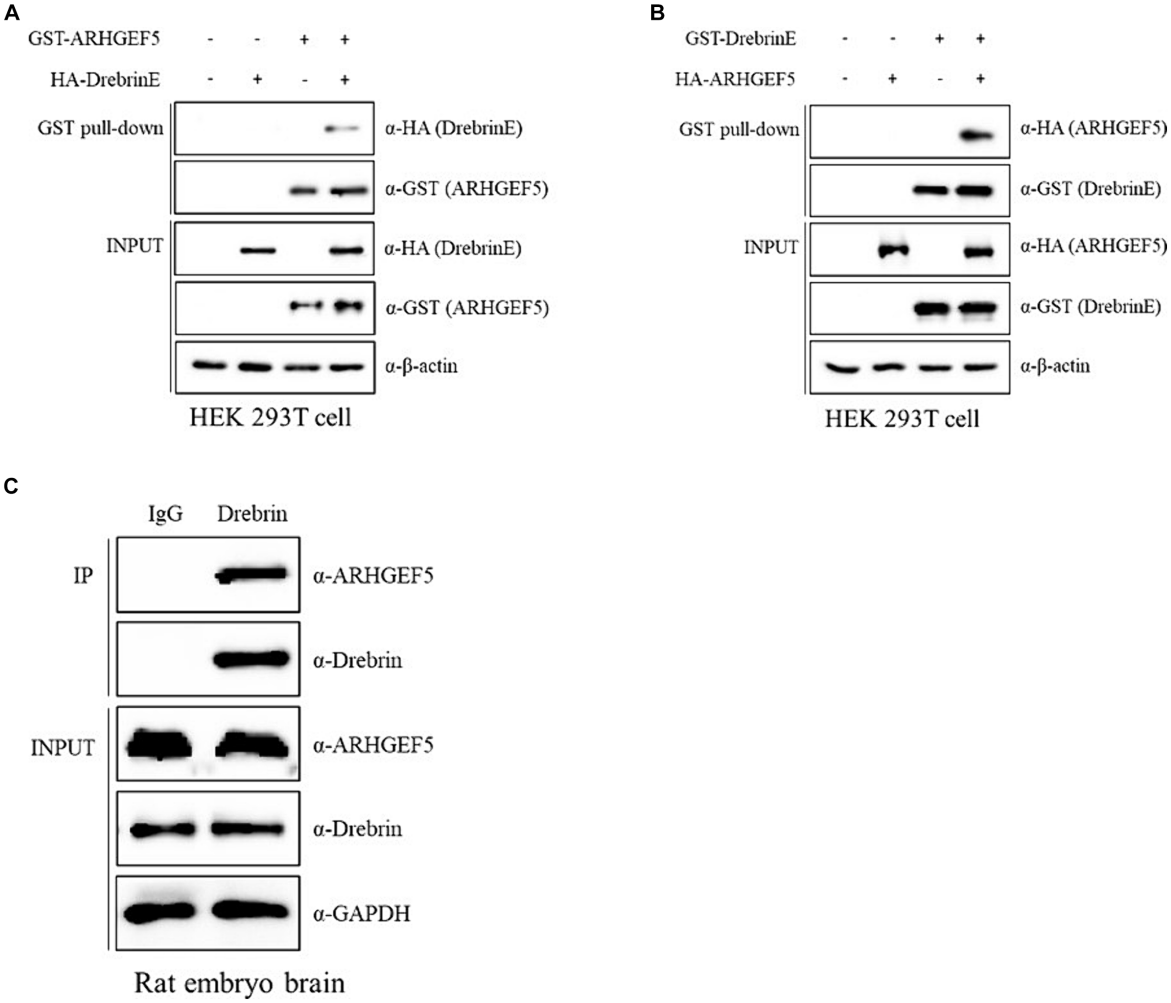
ARHGEF5 interacts with Drebrin in the embryonic brain. **(A,B)** Protein–protein interaction assays were conducted using HEK293T cells transfected with GST- or HA-tagged ARHGEF5 and Drebrin E, followed by a pull-down with glutathione sepharose beads. The bound proteins were detected by immunoblotting with anti-HA and anti-GST antibodies. **(C)** A co-immunoprecipitation assay was conducted using rat embryonic brain lysates with anti-Drebrin antibody. The bound proteins were detected by immunoblotting with anti-ARHGEF5 and anti-Drebrin antibodies.

To validate the interaction between ARHGEF5 and Drebrin E, we conducted pull-down assays using GST- or HA-tagged versions of ARHGEF5 and Drebrin E and demonstrated that HA-Drebrin was pulled down with GST-ARHGEF5 and HA-ARHGEF5 was pulled down with GST-Drebrin (Figures 2A,B). Importantly, the interaction between endogenous ARHGEF5 and Drebrin E was verified by immunoprecipitation assays using rat embryonic brain lysates (Figure 2C). These results suggest that ARHGEF5, together with Drebrin E, may regulate neuronal morphogenesis or migration by affecting both F-actin and microtubules during early brain development.

### 3.3 ARHGEF5 mediates the interaction between Cdk5 and Drebrin

**Figure 3:**
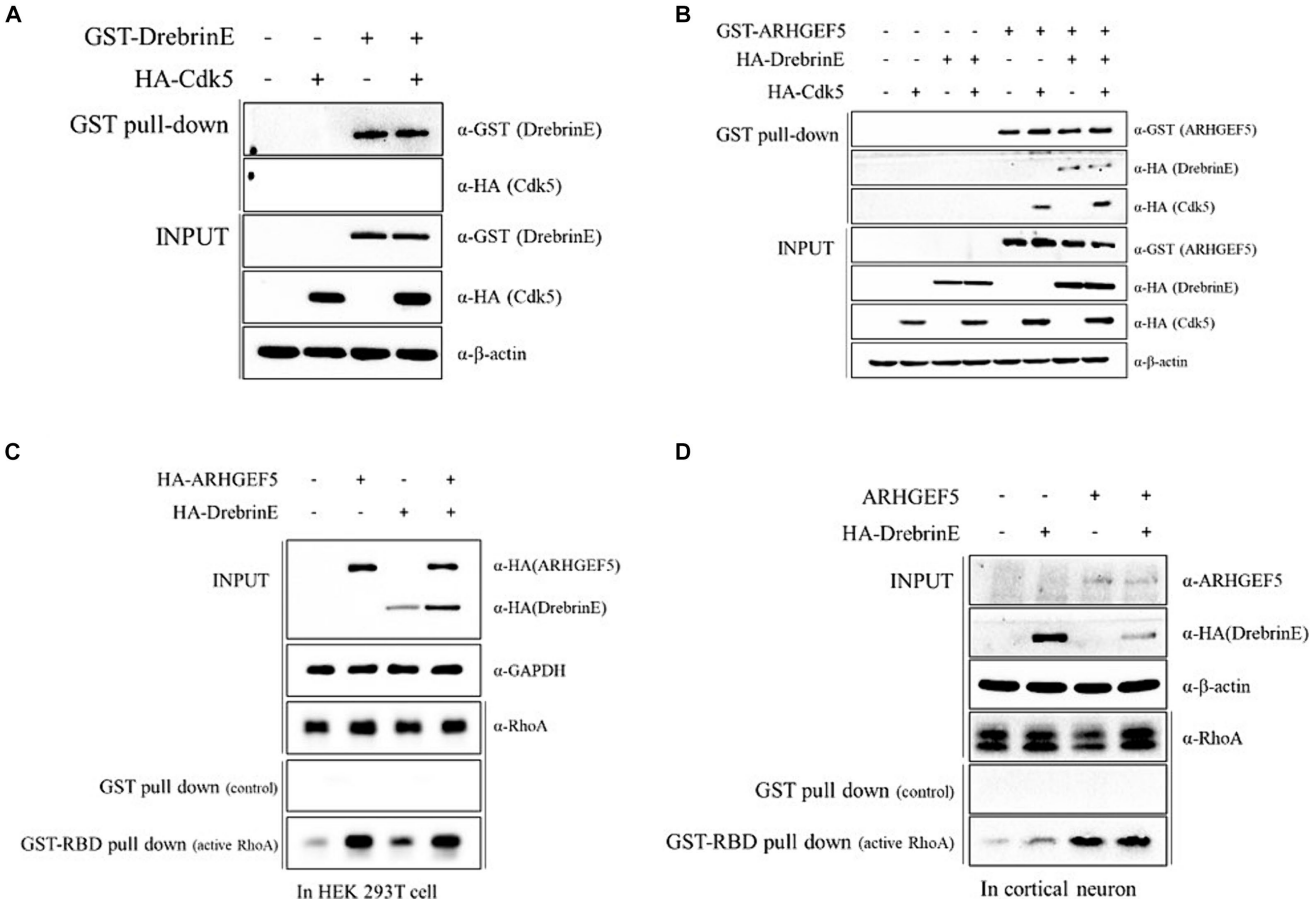
ARHGEF5 mediates the interaction between Cdk5 and Drebrin. **(A,B)** The protein–protein interaction assay was conducted using HEK293T cells transfected with GST-Drebrin E and HA-Cdk5 **(A)** or with GST-ARHGEF5, HA-Drebrin E, and HA-Cdk5 **(B)**, followed by a pull-down with glutathione sepharose beads. The bound proteins were detected by immunoblotting with anti-GST and anti-HA antibodies. **(C,D)** The RhoA activity assay was conducted using HEK293T cells transfected with HA-ARHGEF5 and HA-Drebrin E **(C)** and cultured rat cortical neurons transfected with ARHGEF5 and HA-Drebrin E **(D)**, followed by pull-down with glutathione sepharose beads interacting with the RhoA binding domain (RBD). RhoA was detected by immunoblotting with anti-RhoA antibody.

Considering the role of Cyclin-dependent kinase 5 (Cdk5) in phosphorylating Drebrin during early brain development (Tanabe et al., 2014), we explored whether ARHGEF5 is involved in the interaction between Drebrin E and Cdk5. Pull-down assays showed that Drebrin E alone was unable to co-precipitate Cdk5 (Figure 3A). However, ARHGEF5 was effective in simultaneously co-precipitating Drebrin and Cdk5 (Figure 3B), suggesting ARHGEF5’s essential scaffolding function in bridging the interaction between Cdk5 and Drebrin E.

We then explored whether Drebrin E is involved in ARHGEF5 activation by measuring the amount of active RhoA. Drebrin expression modestly increased active RhoA levels in HEK293T cells and cortical neurons, though not to the extent seen with ARHGEF5 expression alone (Figures 3C,D). The simultaneous expression of Drebrin E and ARHGEF5 did not further enhance RhoA activation beyond ARHGEF5’s sole effect (Figures 3C,D). These data suggest that ARHGEF5 may recruit Drebrin E and Cdk5, with phosphorylated Drebrin E involved in the activation of RhoA by ARHGEF5.

### 3.4 ARHGEF5’s activity is necessary for modulating microtubule organization

**Figure 4:**
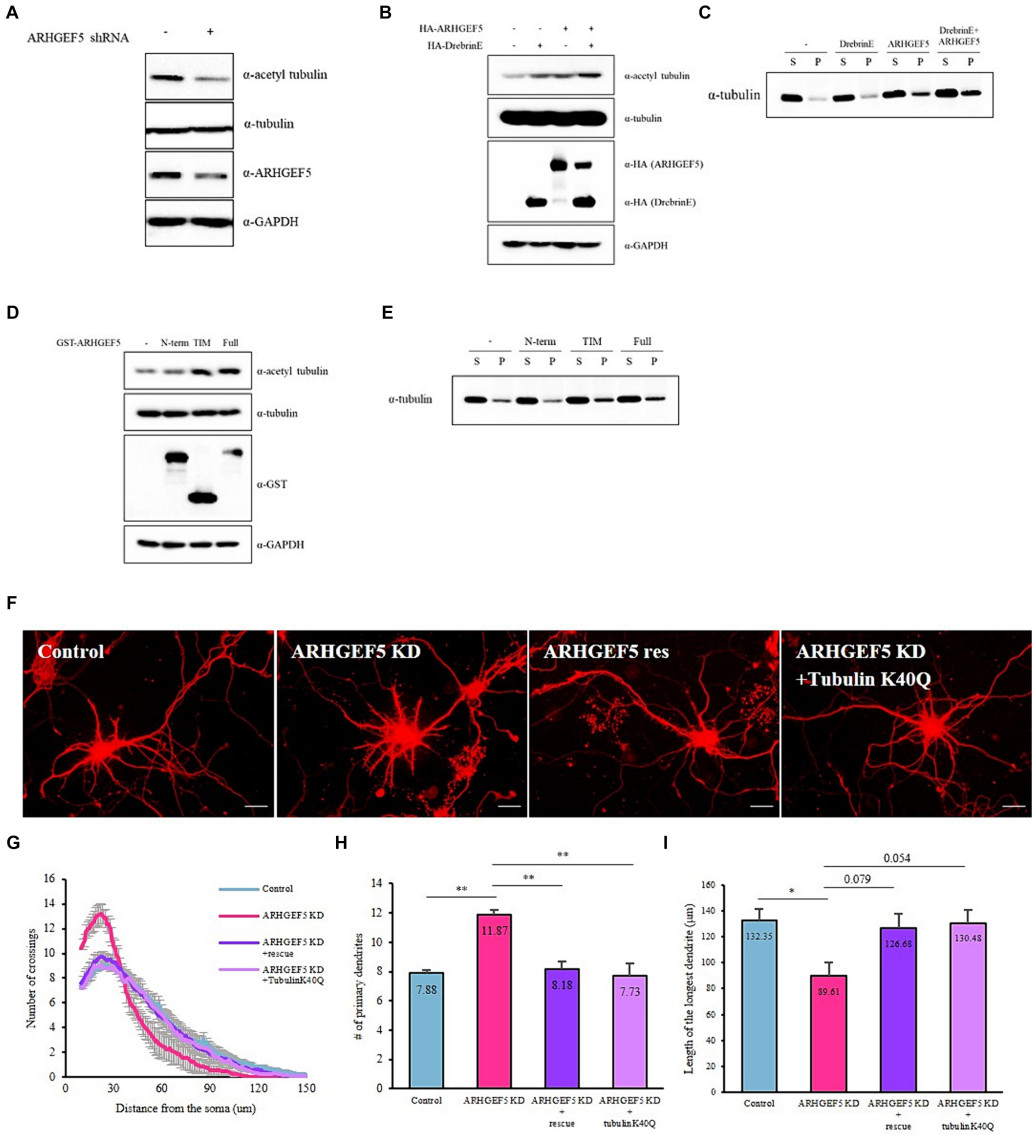
ARHGEF5’s activity is necessary for modulating microtubule organization. **(A)** Immunoblot shows the levels of acetylated α-Tubulin, total α-Tubulin, and ARHGEF5 in HEK293T cells transfected with or without human ARHGEF5 shRNA. GAPDH was used as the loading control. **(B)** Immunoblot shows the levels of acetylated α-Tubulin and total α-Tubulin in HEK293T cells transfected with HA-ARHGEF5 or HA-Drebrin. GAPDH was used as the loading control. **(C)** Immunoblot shows the levels of soluble/polymerized α-Tubulin in HEK293T cells transfected with HA-ARHGEF5 or HA-Drebrin. **(D)** Immunoblot shows the levels of acetylated α-Tubulin and total α-Tubulin in HEK293T cells transfected with GST-tagged ARGHEF5, GST-tagged TIM, or GST-tagged ARHGEF5 N-terminus lacking the TIM domain. GAPDH was used as the loading control. **(E)** Immunoblot shows the levels of soluble/polymerized α-Tubulin in HEK293T cells transfected with GST-ARGHEF5, GST-TIM, or GST-ARHGEF5 N-term. **(F)** Representative images for each group of neurons transfected with ARHGEF5 shRNA or ARHGEF5 shRNA together with shRNA-resistant ARHGEF5 construct or α-Tubulin K40Q construct. **(G)** Quantification of dendritic complexity using Sholl analysis. **(H)** The number of primary dendrites. **(I)** Length of the longest dendrite. *n* > 30 from three independent experiments for each group. All data are presented as mean ± SEM. ^*^p < 0.05, ^**^p < 0.01, ^***^p < 0.001 determined by one-way ANOVA with Tukey’s *post hoc* test.

Drebrin directly interacts with F-actin bundles and facilitates the polymerization of microtubules via its interaction with EB3 (Merriam et al., 2013). We explored ARHGEF5’s involvement in this context by assessing its effect on acetylated α-tubulin and polymerized α-tubulin levels, well-established markers of microtubule stability (Schulze et al., 1987). ARHGEF5 knockdown led to a decrease in acetylated α-tubulin levels (Figure 4A), while its overexpression, particularly when combined with Drebrin E, enhanced the levels of acetylated and polymerized α-tubulin (Figures 4B,C). These findings suggest ARHGEF5, potentially in conjunction with Drebrin E and Cdk5, plays a key role in microtubule organization by influencing the level of acetylated α-tubulin.

ARHGEF5 is composed of a long N-terminus domain whose function is still unknown and a TIM domain that possesses guanine nucleotide exchange factor (GEF) activity. To determine if ARHGEF5’s GEF activity is crucial for microtubule organization, we measured the levels of acetylated and polymerized α-tubulin in cells expressing full-length ARHGEF5, its N-terminus (missing the TIM domain), or the TIM isoform, which includes the GEF domain. Results showed enhanced acetylation and polymerization of α-tubulin with the full-length ARHGEF5 and the TIM isoform, not observed with the N-terminus alone (Figures 4C,D), underscoring the significance of the ARHGEF5’s GEF domain in regulating microtubule organization.

Given the role of α-tubulin acetylation in neuronal morphogenesis (Jenkins et al., 2017; Dan et al., 2018; Niu et al., 2023), we investigated if expressing an α-tubulin-Lys40-acetylation mimetic mutant (K40Q) could mitigate the morphogenesis defects observed in ARHGEF5 knockdown neurons. Co-transfection with the K40Q mutant in cultured hippocampal neurons reversed the ARHGEF5 knockdown phenotype (Figure 4F), indicating ARHGEF5’s essential role in α-tubulin acetylation and proper microtubule organization crucial for neuronal development.

### 3.5 ARHGEF5 is essential for proper neuronal migration

**Figure 5:**
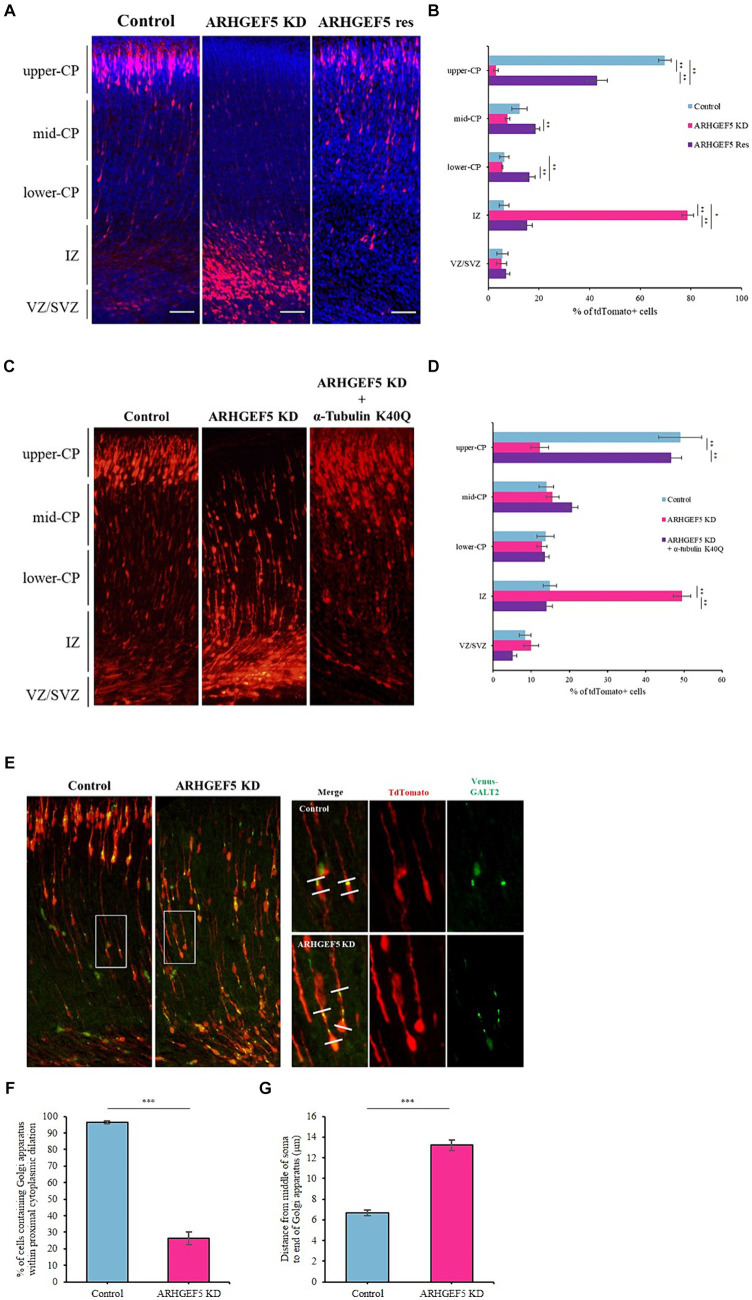
ARHGEF5 is essential for proper neuronal migration. **(A)** Representative images for each group of embryonic brains electroporated with rodent ARHGEF5 shRNA or ARHGEF5 shRNA plus shRNA-resistant ARHGEF5 construct at E14.5 and fixed at E18.5. Nuclei were stained with Hoechst 33342. **(B)** Quantification of the population of tdTomato-positive cells in indicated regions of the cortex. CP, cortical plate. IZ, intermediate zone. VZ/SVZ, ventricular zone/sub-ventricular zone. *n* = 6 for each group. **(C)** Representative images for each group of embryonic brains electroporated with rodent ARHGEF5 shRNA or ARHGEF5 shRNA plus α-Tubulin K40Q construct at E14.5 and fixed at E18.5. **(D)** Quantification of the population of tdTomato-positive cells in indicated regions of the cortex. *n* = 4 for each group. **(E)** Representative images for each group of embryonic brains electroporated with rodent ARHGEF5 shRNA plus Venus tagged GALT2 at E14.5 and fixed at E18.5. **(F)** Quantification of the population of cells containing Venus-GALT2 within proximal cytoplasmic dilation. *n* = 136 for control, *n* = 182 for ARHGEF5 shRNA from 4 brains. **(G)** Quantification of Golgi dispersal. Distance from the middle of the soma to the end of Venus-GALT2 signal. *n* = 134 for control, *n* = 161 for ARHGEF5 shRNA from 4 brains. All data are presented as mean ± SEM. ^*^p < 0.05, ^**^p < 0.01, ^***^p < 0.001 determined by one-way ANOVA with Tukey’s *post hoc* test **(B,D)** and unpaired two-tailed Student’s t-test **(F,G)**.

Cortical neurons, originating from radial glial stem cells in the ventricular zone, migrate towards the cortical plate (CP) during embryonic stages E14.5 to E18.5 (Nadarajah and Parnavelas, 2002). Recognizing the importance of α-tubulin acetylation in neuronal migration (Creppe et al., 2009; Li et al., 2012), we explored ARHGEF5’s necessity in this process. After introducing ARHGEF5 shRNA via *in utero* electroporation at E14.5, we observed significant retention of ARHGEF5-knockdown neurons in the intermediate zone by E18.5, contrasting with the successful migration of control neurons. This migration defect was rescued by co-transfection with shRNA-resistant ARHGEF5 (Figures 5A,B). Then, we investigated whether the acetylation mimetic mutant, α-tubulin-K40Q, could mitigate the defect caused by ARHGEF5-knockdown. As shown in Figures 5C,D, the impaired migration was rescued by the expression of α-tubulin-K40Q. This indicates ARHGEF5’s role in α-tubulin acetylation during cortical neuron migration.

Neuronal migration requires dynamic cytoplasmic dilation, where the centrosome and Golgi apparatus are strategically positioned for effective movement (Bellion et al., 2005; Schaar and Mcconnell, 2005; Nishimura et al., 2014; Vinopal et al., 2023). Microtubule dynamics is a crucial factor for Golgi apparatus positioning and nucleokinesis during neuronal migration (Rivas and Hatten, 1995; Nishimura et al., 2014; Goo et al., 2023). Microtubule destabilizers, such as Nocodazole, decreased the acetylated α-tubulin levels and dispersed compact Golgi apparatus in cultured hippocampal neurons (Meseke et al., 2013). Therefore, we explored the role of ARHGEF5 in this context by using ARHGEF5 knockdown shRNA and Venus-tagged GALT2 for *in utero* electroporation at E14.5. Contrary to control neurons where the Golgi apparatus localized in the cytoplasmic dilation at the proximal end of the leading process, ARHGEF5 knockdown resulted in Golgi dispersal towards the distal end of the leading process (Figures 5E,F). Taken together, these results indicate that ARHGEF5 is essential for neuronal migration during early brain development by contributing to α-tubulin acetylation, which is necessary for microtubule organization suitable for neuronal migration.

## 4 Discussion

During brain development, neurons originating in the ventricular zone migrate to their destined positions, forming the cerebral cortex’s specific layered structure. This process is essential for the cortex’s functionality, with subsequent dendritic development. This study reveals significant findings regarding the role of ARHGEF5 in neuronal development. Knockdown of *Arhgef5* resulted in aberrant dendritic development in cultured neurons and notable defects in neuronal migration within the cerebral cortex. These defects were alleviated by the expression of acetylated α-tubulin, indicating an association of ARHGEF5 with microtubule dynamics in neurons.

The human *Arhgef5* gene is located in the human chromosome 7q33-q35 (Takai et al., 1995). Human disease database MalaCards^2^ suggests a possibility that ARHGEF5 may be associated with Williams-Beuren syndrome, a rare genetic disorder caused by the deletion of the genetic material of chromosome 7, characterized by developmental delay, moderate intellectual disability, cardiovascular problems, short stature, and distinctive facial features. Additionally, deletions in the chromosome 7q33-q35 region have been linked to autism spectrum disorders, unexplained developmental delays and intellectual disability, significant language delays, and microcephaly (Rossi et al., 2008; Sehested et al., 2010; Dilzell et al., 2015; Kale and Philip, 2016; Gurkan et al., 2020). Given the uncertain nature of ARHGEF5’s link to these conditions, its exact role in brain and neuronal development is yet to be defined. This ambiguity necessitates dedicated research to uncover how ARHGEF5 may influence the brain’s structural development and functional maturation.

In this investigation, we demonstrate that ARHGEF5 is expressed during the initial phases of cortical neuron development (Figure 1), and it engages in interaction with Drebrin E, an isoform prevalent in the embryonic brain (Figure 2). Drebrin, facilitating crosstalk between F-actin and microtubule (Shirao and Sekino, 2017), plays multifaceted roles in neuronal development, including regulation of neurite outgrowth, formation of dendritic branches, and synaptic plasticity (Geraldo et al., 2008; Koganezawa et al., 2017; Shirao et al., 2017). Moreover, Drebrin is required for proper neuronal migration of GnRH neurons, hippocampal granule cells, neuroblasts, oculomotor neurons, and cerebellar granule neurons (Dun et al., 2012; Sonego et al., 2015; Trivedi et al., 2017; Shan et al., 2021). Drebrin is phosphorylated by Cyclin-dependent kinase 5 (Cdk5), which promotes Drebrin’s activity and subcellular localization (Worth et al., 2013; Tanabe et al., 2014; Gordon-Weeks, 2016), but the precise mechanisms involved remain to be fully elucidated. Our data showing that ARHGEF5 can co-precipitate with both Drebrin E and Cdk5, but that Drebrin E does not associate with Cdk5 in the absence of ARHGEF5 (Figure 3), suggest that ARHGEF5 could serve as a scaffold, enhancing Drebrin’s phosphorylation by facilitating its interaction with Cdk5. The phosphorylation of Drebrin at Serine 142, located within the interaction region of the BB and CC domains, alleviates the auto-inhibitory interaction that otherwise limits F-actin bundling and EB3 binding (Gordon-Weeks, 2016). Pull-down assays have shown that the C-terminal domain of ARHGEF5 binds to Drebrin’s N-terminal region, which includes the CC domain (Supplementary Figure S2), suggesting Drebrin’s interaction with ARHGEF5 may enhance its phosphorylation by Cdk5. Further investigation is required to precisely define ARHGEF5’s role in modulating Cdk5’s phosphorylation of Drebrin.

Previous studies revealed that ARHGEF5 regulates actin cytoskeletal dynamics by activating the RhoA-ROCK pathway, thereby promoting the migration and invasion of cancer cells (Kuroiwa et al., 2011; Lee et al., 2015; Komiya et al., 2016). In this study, we discovered that ARHGEF5 not only plays a role in RhoA activation but also contributes to microtubule stability via α-tubulin acetylation (Figure 4). Since RhoA regulates both actin filaments and microtubules via ROCK or mDia (Govek et al., 2005), it’s plausible that ARHGEF5 influences microtubule stability through these pathways. ROCK is known to induce microtubule destabilization by phosphorylating microtubule-associated proteins, including MAP2, Tau, CRMP2, and Doublecortin, resulting in their release from microtubules (Schofield et al., 2012). Conversely, mDia stabilizes microtubules by forming complexes with microtubule plus-end binding proteins and capping the microtubules (Wen et al., 2004). Moreover, RhoA-mDia pathway regulates Golgi deployment in neurons (Hong et al., 2018). Since our data indicate that ARHGEF5 binds to Drebrin, which is known to interact with mDia (Ginosyan et al., 2019) and regulates the positioning of Golgi apparatus, we hypothesize that ARHGEF5’s effect on microtubule stability may be mediated through the RhoA-mDia pathway rather than the RhoA-ROCK pathway. The precise mechanisms behind this regulation and α-tubulin acetylation remain to be explored.

The intricate coordination between actin filaments and microtubules is crucial for neurons to reach their target locations during brain development. Previous research has demonstrated that in migrating neuroendocrine gonadotropin-releasing hormone (GnRH) cells, anchoring of microtubule plus-ends to the cortical actin within the leading process facilitates the nucleokinesis (Hutchins and Wray, 2014). Moreover, actomyosin contraction in the proximal leading process is vital for positioning the Golgi apparatus and forward movement of centrosome and soma (Solecki et al., 2009; Trivedi et al., 2014). Drebrin, a protein that links actomyosin to microtubules within the proximal leading process, plays a significant role in the forward movement of microtubules and soma in migrating cerebellar granule neurons (Trivedi et al., 2017). Thus, exploring the potential involvement of ARHGEF5 in actomyosin contractility during neuronal movement or development would be an interesting future research topic.

In conclusion, our research underscores the essential role of ARHGEF5 in early neuronal development, particularly its critical function in regulating the cytoskeletal dynamics vital for neuronal migration and morphogenesis. The interaction between ARHGEF5 and Drebrin E, along with ARHGEF5’s impact on α-tubulin acetylation, highlights its pivotal role in regulating microtubule stability, thereby identifying it as a fundamental regulator in neuronal development. This study establishes a foundation for further exploration of ARHGEF5’s mechanistic contributions to neuronal development, paving the way for enhanced understanding of its role in guiding neurodevelopmental processes.

## Data availability statement

The raw data supporting the conclusions of this article will be made available by the authors, without undue reservation.

## Ethics statement

The animal study was approved by Institutional Animal Care and Use Committee of Chungnam National University. The study was conducted in accordance with the local legislation and institutional requirements.

## Author contributions

J-yeK: Conceptualization, Formal analysis, Funding acquisition, Investigation, Methodology, Writing – original draft. H-GH: Data curation, Formal analysis, Investigation, Writing – original draft, Conceptualization, Writing – review & editing. H-JJ: Investigation, Writing – review & editing. SK: Formal analysis, Methodology, Writing – review & editing. M-kK: Conceptualization, Methodology, Writing – review & editing. J-YK: Conceptualization, Funding acquisition, Supervision, Writing – review & editing, Data curation, Writing – original draft.

## Footnotes

^1^ https://www.proteinatlas.org/ENSG00000050327-ARHGEF5/brain

^2^ https://www.malacards.org/
