# Supplementary material for: ARHGEF5 binds Drebrin and affects α-tubulin acetylation to direct neuronal morphogenesis and migration during mouse brain development
Source: Front Mol Neurosci. 2024 Jun 12;17:1421932. doi: 10.3389/fnmol.2024.1421932 (PMC11199874; doi:10.3389/fnmol.2024.1421932)
Supplement: Supplementary file 1 [file Data_Sheet_1.DOCX]

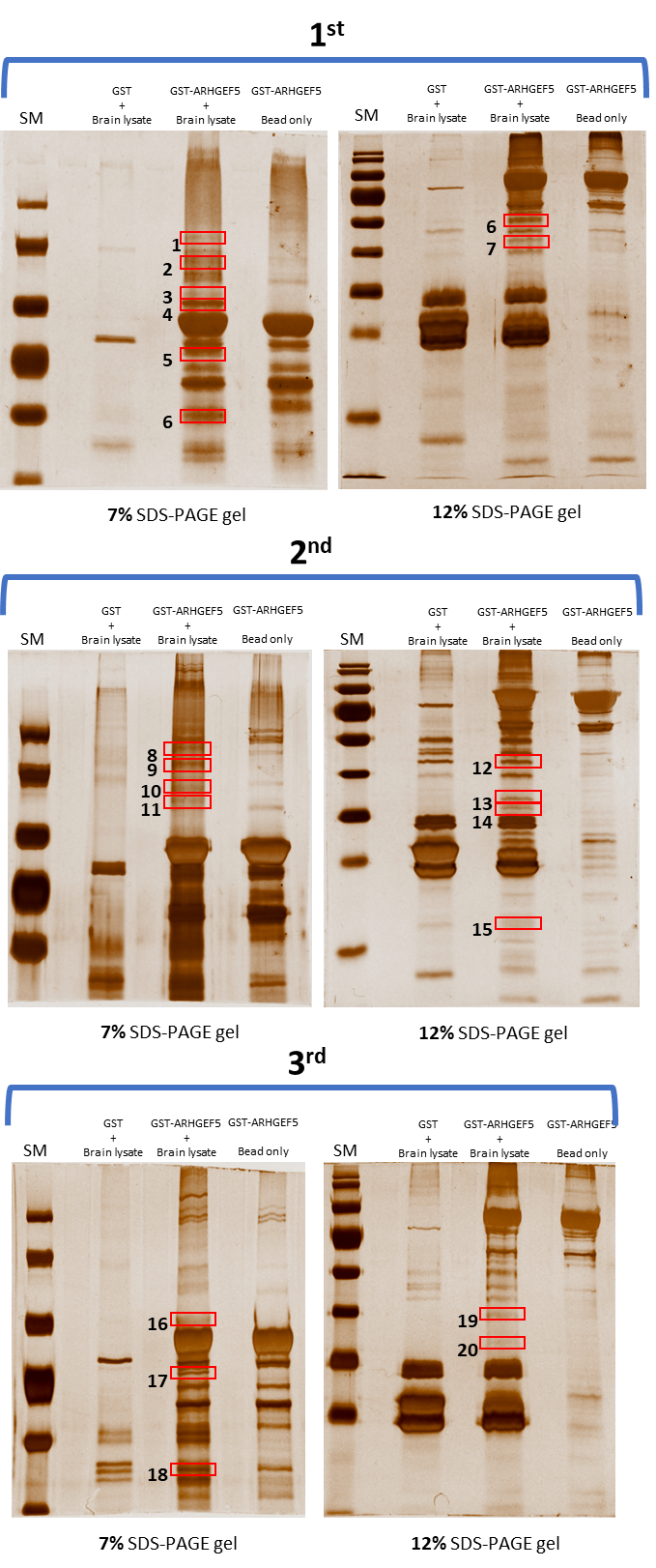


**B**

**A**

**Supplementary Figure 1. Screening of ARHGEF5 interactors in rat embryonic brain lysates.** **(A)** The silver staining results show distinct protein bands exclusively associated with glutathione sepharose beads bound to GST-ARHGEF5 (lane 2). The protein bands observed on glutathione sepharose beads bound to GST with brain lysates (lane 1) or glutathione sepharose beads bound to GST-ARHGEF5 without brain lysates (lane 3) served as negative controls. Three independent experiments were conducted. The indicated 20 bands were analyzed by LC-MS/MS. **(B)** The identified protein list.


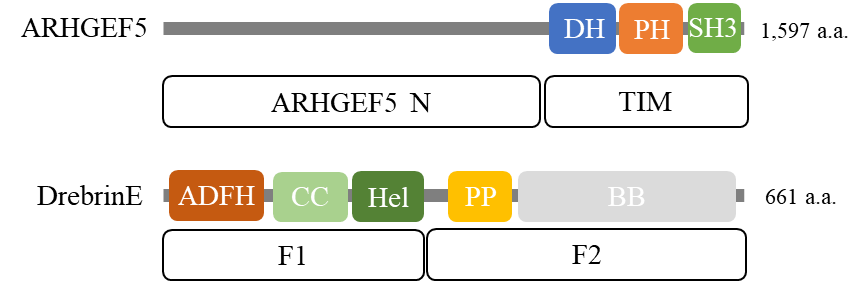

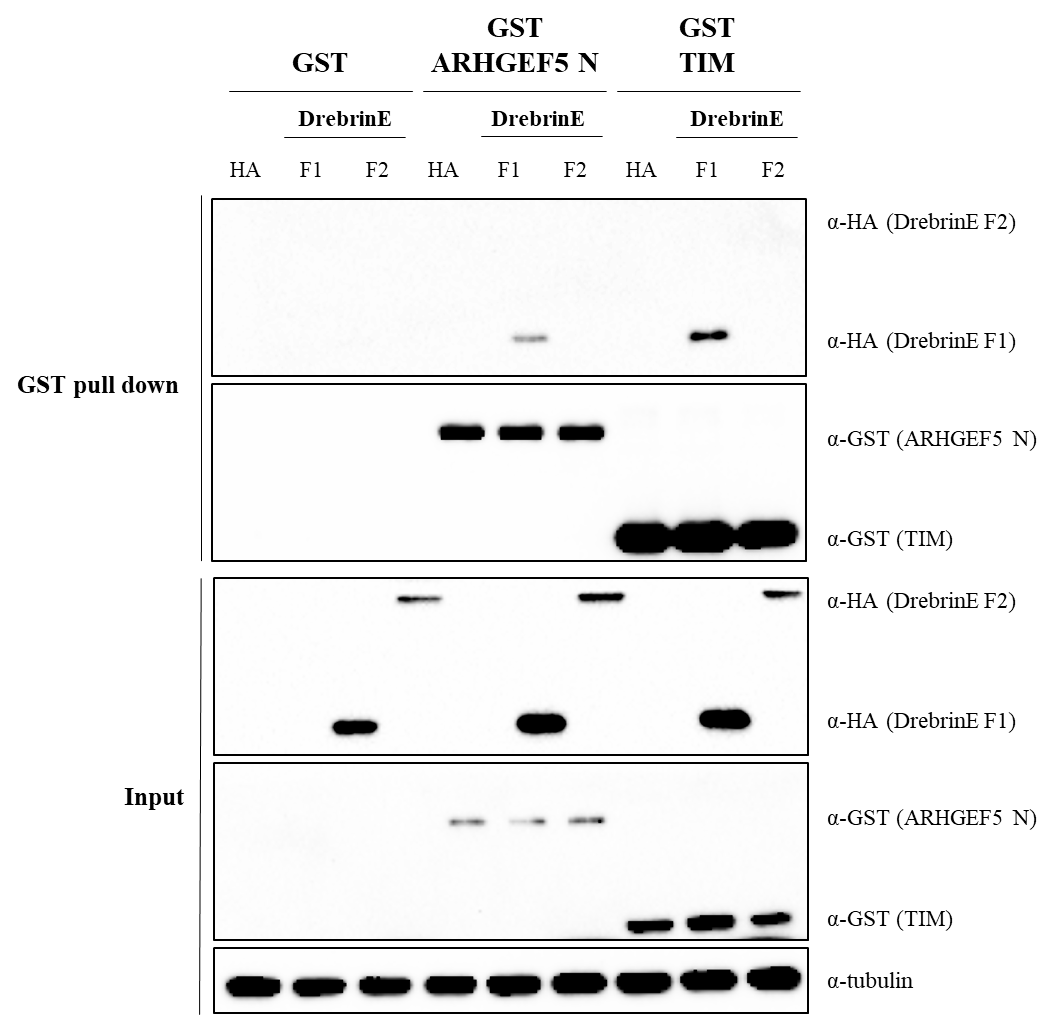

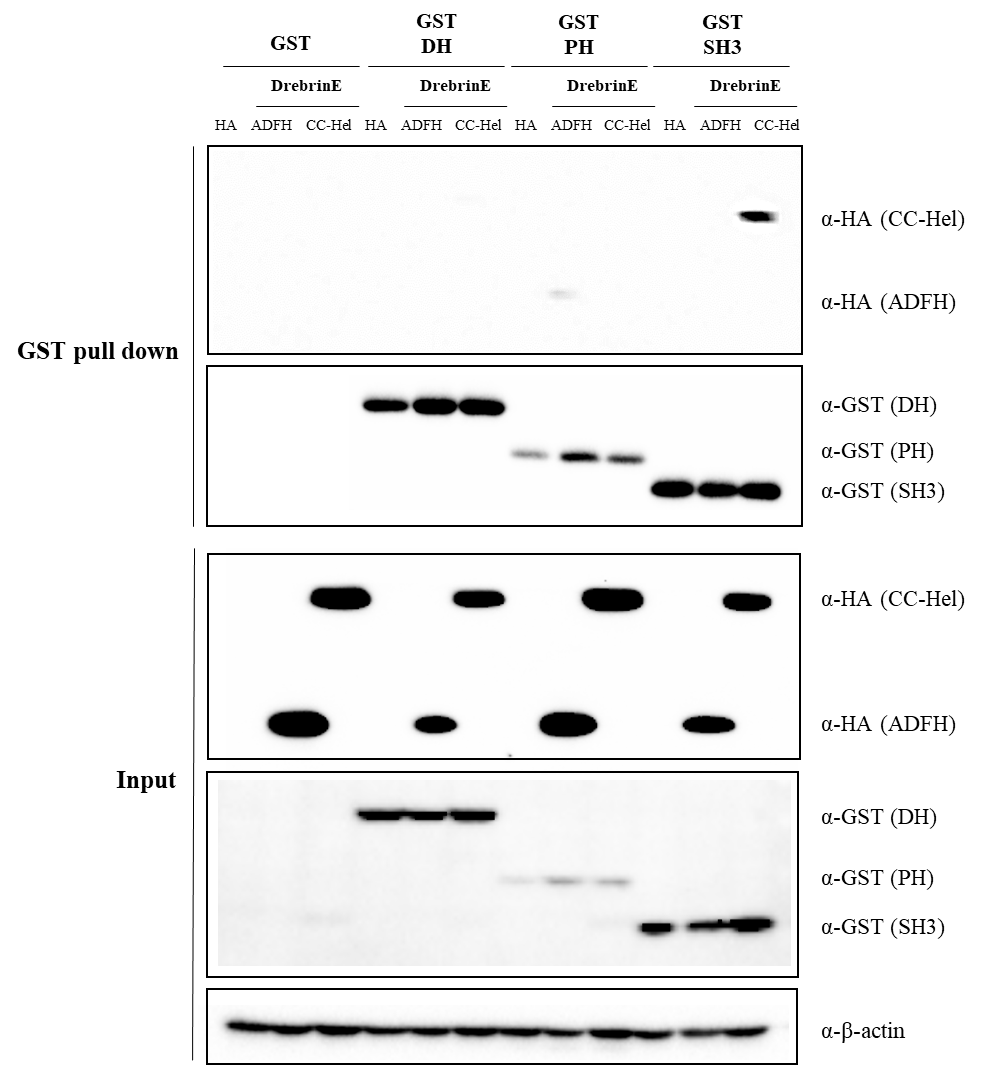


**A**

**B**

**C**

**Supplementary Figure 2. ARHGEF5 interacts with N-terminus of DrebrinE**. **(A)** The functional domains of ARHGEF5 and DrebinE. **(B)** The protein-protein interaction assay was conducted using HEK293T cells transfected with HA-DrebrinE F1 or HA-DrebrinE F2 together with GST, GST-ARHGEF5 N-term, or GST-TIM, followed by pull-down with glutathione sepharose beads. The bound proteins were detected by immunoblotting with anti-GST and anti-HA antibodies. **(C)** The protein-protein interaction assay was conducted using HEK293T cells transfected with HA-DrebrinE ADFH or HA-DrebrinE CC-Hel together with GST, GST-ARHGEF5 DH, GST-ARHGEF5 PH, or GST-ARHGEF5 SH3, followed by pull-down with glutathione sepharose beads. The bound proteins were detected by immunoblotting with anti-GST and anti-HA antibodies.
